# Occupation-Related Biological Health Hazards and Infection Control Practices among Indian Veterinarians

**DOI:** 10.1155/2022/2503399

**Published:** 2022-02-24

**Authors:** Rajendra Palkhade, SukhDev Mishra, Sukhadeo Barbuddhe

**Affiliations:** ^1^ICMR-National Institute of Occupational Health, Meghaninagar, Ahmedabad, Gujarat, India; ^2^ICAR-National Research Centre on Meat, Chenicherla, Hyderabad, India

## Abstract

Veterinarians experience different types of health hazards from their occupation. Studies on the prevalence and occurrence of biological health hazards in veterinary medicine in India are scant and probably underreported. Thus, we sought to assess the biological health hazards and infection control practices (ICPs) among veterinarians from the states of Gujarat and Maharashtra, India. A cross-sectional survey was conducted among veterinarians (*n* = 562) from Gujarat and Maharashtra states in India to identify biological health hazards and ICPs for the prevention of occupational health hazards during 2016–2017 by personally contacting them. Responses regarding a biological hazard and ICPs were recorded. Descriptive analysis was attempted, and continuous variables are presented as the mean ± SD. Categorical variables are reported as counts and percentages (%). Most of the veterinarians (49.3%) worked in the field and were continuously exposed to different types of biological health hazards, especially zoonoses, ranging from mild and self-limiting to fatal diseases (e.g., brucellosis (subclinical and clinical form) and rabies (fatal)) without common prophylactic vaccinations, such as rabies and tetanus. While inquiring medical health status of the veterinarians, only 35.8% of the total respondents underwent a routine medical health checkup within the past year, and 56.9% did not receive a routine dose of an anthelmintic for deworming. Forty-nine percent of the respondents took all necessary precautions, including wearing an apron, facemask, and gloves. In contrast, 10.2% of the respondents wore only an apron, and 8.4% of respondents did not take any precautions while performing their day-to-day work. In total, 40.2% of the respondents followed the proper method of handwashing, that is, washing hands between patient examinations. In contrast, 27.9% of the respondents washed their hands once after completing the work. The majority of the respondents (87.7%) reported an urgent need for occupational hazards and safety (OHS) training in continued veterinary education (CVE) programs. The present study demonstrates that veterinarians in the states of Gujarat and Maharashtra in India pay less attention to their own health that may increase the risk of occupation-related biological health hazards. These results suggest that safety and ICPs are not prioritized, which are serious concerns. These findings may be useful for developing policies to prevent occupationally related biological health hazards among veterinarians in India.

## 1. Introduction

Veterinarians play a crucial role in the development, well-being, health care, and welfare of animals and in upgrading the rural economy of India by empowering socioeconomic classes. The main components of the job profile of veterinarians in India include prevention, treatment, management of animal diseases, improvement of production, and extension activities to implement government schemes to elevate the socioeconomic class through animal husbandry activities. Most farmers in rural areas receive veterinary health care services from government-appointed veterinarians [1]. Veterinarians also contribute to public health and protect humans against zoonotic diseases by using their scientific knowledge and technical skills. While performing their day-to-day work, veterinarians come in close contact with various sick animals and birds, and some animals are carriers of infections despite appearing healthy. Such apparently healthy animals also pose a risk of transmitting various zoonotic diseases to veterinarians [2]. Zoonoses are diseases and infections that are naturally transmitted between vertebrate animals and humans [3]. Due to the virtue of their occupation, veterinarians are exposed to different occupational health hazards, viz., physical, chemical, biological [4, 5], and psychological hazards. Veterinarians are exposed to possible carcinogens, including radiation, pesticides, anesthetic, and zoonotic infections, including viruses [6]. Occupational health and safety (OHS) is often focused on workers involved in manufacturing and construction, but infectious diseases often more frequently occur in occupational settings [7].

Veterinarians are at high risk for occupation-related biological health hazards, especially in countries that exercise traditional farming activities, where keeping backyard livestock remains customary, and there is low knowledge of zoonoses among livestock keepers [8].

Veterinarians in Australia are considered a high-risk group for occupational hazards [4]. Approximately 75% of emerging human infectious diseases are directly or indirectly linked to animal origins [9]. This finding highlights the importance of identifying occupational health hazards among veterinarians.

The most commonly reported zoonotic diseases in veterinarians include ringworm [10, 11]; cat scratch fever, which is also known as Bartonellosis and caused by *Bartoneolla henselae*; salmonellosis [10, 12]; brucellosis [13, 14]; leptospirosis [15]; and Q-fever [16, 17]. Despite the risk of different zoonotic diseases in the field of veterinary medicine, a recent study conducted among United Arab Emirates' large-animal (39%) and small-animal veterinarians (41%) showed a failure to use personal protective equipment (PPE) while handling blood samples [18].

Complete elimination of the potential of contracting zoonotic diseases is an unrealistic goal; thus, the focus should be on preventing diseases by decreasing the risk. New activities and advanced technologies have generated new zoonotic and occupational risks. The Indian subcontinent is one of the four global hotspots at increased risk for the emergence of new infectious diseases [19]. Therefore, the identification/evaluation of biological hazards among Indian veterinarians is extremely important.

The risk of spreading zoonotic infections has always represented a threat to public health in terms of productive and economic losses, and veterinarians may act as carriers. To the best of our knowledge, comprehensive studies have not been conducted to date to identify occupation-related biological health hazards and ICPs among Indian veterinarians. There is an urgent need to assess occupational hazards in veterinary practice to develop strategies for prevention. Hence, the objectives of the present survey was to assess biological health hazards and ICPs among veterinarians in the states of Gujarat and Maharashtra, India, which can help to encourage science-based biological health hazard prevention policies for veterinarians and associated workers.

## 2. Materials and Methods

This study was conducted among veterinarians (*n* = 562) in the states of Gujarat and Maharashtra, India. The survey tool (a set of questionnaires) was designed by reviewing the internationally conducted studies [10, 20, 21] and [22] on related topics of survey objectives. Experts in this profession were consulted to identify different occupation-related biological health hazards and ICPs. The questionnaire was modified thereafter on the feedback received from the pre-test conducted among veterinarians, which was not included in the survey analysis. Veterinarians were included in the study if they fulfilled the inclusion criteria and were willing to participate. During 2016–2017, the self-assessed questionnaire was completed by veterinarians who were personally contacted. Prior to the inclusion of individual veterinarians in the study, the purpose of the study was described to the veterinarians, and written consent was obtained. Only veterinarians with recent veterinary work experience for at least 2 years and those who were continuously working despite being retired from service were included in the study. Retired (nonpracticing) veterinarians, veterinarians with less than 2 years of the experienced, nonclinical academic staff of veterinary colleges/universities, and veterinarians involved in administrative/extension activities were excluded from the study. All questions were of the close-ended type, and the participants were asked to rank or select from a list of provided options.

Recall for each response in the present study was limited to occurrences within the last 2 years. Data were collected on biological health hazards and ICPs based on the following categories:

1. Individual health: prophylactic vaccination, routine medical health checkup, screening for zoonotic diseases, deworming doses, any ailments/allergy, and medical treatment sought at the time of the interview.
2. Public health: sharing and discussion of zoonosis among subordinates, clients, farmers, general population, and physicians.
3. ICPs: handwashing pattern, use of PPE, awareness about different types of occupational health hazards (physical, chemical, and biological hazards and allergies) in the field of veterinary medicine, and source(s) of their information about occupational hazards and risk.

For sample size estimation, we assumed *p* = 0.05 with the desired precision of 5% and a confidence level of 95% as suggested by [23] in case of unavailability of suitable evidence [23].

The sample size was calculated using the formula *n* ≥ *p*(1 − *p*) ∗ (*Z*/*d*)^2^, where *d* is the desired precision. The study required a minimum sample size of 427 subjects considering a 10% nonresponse rate; however, to increase the precision of the study results, we were able to collect and analyze the responses from 562 subjects on the study questionnaire.

The study was approved by the Institutional Human Ethical Committee at ICMR-National Institute of Occupational Health, Ahmedabad (Agenda No. 3.6/2015).

All recorded questionnaire data were transcribed into customized databases using data entry screens in the Epi-Info 7 software. The data were doubly entered to minimize any entry errors. All data errors were reconciled by referring to paper-based data entry. Continuous variables, such as age, are presented as the mean ± SD, and all other attributes of a categorical nature are reported as counts (*n*) and percentages (%). The 95% confidence interval was performed for reported data. All the statistical analysis was performed using *R* Software version 4.

## 3. Results

The results illustrated that the mean age of the veterinarians working in Gujarat and Maharashtra state was 39.94 ± 9.75 years, and most of them worked for more than 50 hours a week.

The majority (80.9%) of the veterinarians agreed that they have suffered needlestick injuries in the last 2 years. Needlestick injuries are related to physical hazards, such as local inflammation, and subsequent biological hazards. Hence, the material present in the needle at the time of the puncture event was assessed. Most respondents (60.1%, 28.5%, and 3.4%) reported that needles contained nonspecific drugs, antibiotics, and vaccines (rabies and *brucella* vaccine), respectively, at the time of the needle puncture event (Table 1).

Data were collected on individual health. Only 35.8% of the total respondents had undergone a routine medical health checkup within the past year, and 36.4% of respondents had never undergone a routine medical health checkup. Despite being a high-risk occupational group and constantly being surrounded by animals with increased chances of parasitic infestation, approximately 56.9% of the respondents did not receive a routine dose of an anthelmintic for routine deworming. In contrast, 18.9%, 12.5%, and 11.8% (total 43.2%) of the respondents received an anthelmintic dose within 12, 6, and 4 months, respectively. Among the respondents, only 27.0% underwent screening for zoonotic infections. Total of eight individuals were found positive for brucellosis (for brucellosis screening, different tests were mentioned by the respondents, such as Rose Bengal plate test (RBPT), IgM, and IgG antibiotics detection through ELISA), and one individual was positive for ringworm infections. Half of the respondents (49.1%) did not report any ailments at the time of the study, whereas the remaining respondents suffered from different ailments such as anxiety (9.5%), diabetes (4.7%), enteric disorders (6.4%), hypotension or hypertension (14.9%), and allergies (10.4%). Regarding allergies, the veterinarians suffered from different types of allergies such as fur/dandruff (13.1%), latex gloves (1.6%), chemicals/drugs (4.9%), and vaginal secretions (1.6%). Moreover, 12.5% of the respondents suffered from allergies so severe that they sought medical attention. Half of the surveyed population (49.3%) did not receive any prophylactic vaccinations, such as rabies and tetanus. A total of 39.5% and 11.1% of the respondents received rabies and tetanus vaccines, respectively (Table 2).

Regarding ICPs, approximately half of the respondents (49.0%) were taking all necessary precautions while dealing with animals, including wearing aprons, facemasks, and gloves. In contrast, 10.2% of the respondents wore only an apron, and 8.4% of the respondents routinely worked in the field without taking any precautions. Regarding handwashing, 40.2% of the respondents followed proper handwashing, that is, washing hands between patient examinations, whereas 27.9% of the respondents only washed their hands once after finishing their work day. The practice of washing hands before and after work was followed by 11.4% of respondents and 6.2% of the respondents only washed their hands before eating (Table 3).

Regarding professional collaboration, 70.2% and 23.2% of the respondents were never and rarely contacted by physicians, respectively. Moreover, 55.6% and 36.5% of the respondents never or rarely contacted physicians, respectively, regarding zoonotic diseases (Table 4).

Regarding knowledge about OHS, approximately 35% (34.9%) and 19.9% of the total respondents interviewed were aware of physical and biological health hazards in the veterinary profession, whereas 3.1% and 3.6% were aware of chemical hazards and allergies, respectively. Meetings/training (38.8%) followed by the Internet (39.9%), news bulletins (14.2%), and conferences (7.1%) were reported as tools that played an important role in keeping veterinarians updated about OHS. Most of the respondents (87.7%) expressed an urgent need for the inclusion of OHS training in continuing veterinary education (CVE) programs. Half of the respondents (56.6%) discussed zoonosis occasionally, 6.5% weekly, 13.6% daily, and 12.6% several times a day among their subordinates and client population (Table 5).

## 4. Discussion

There are three main important issues related to occupational health and management: individual health, workplace culture (ICPs), and physical issues. In this study, we focused on the individual health and workplace culture among veterinary medicine professionals in the states of Gujarat and Maharashtra, India, to explore the biological health hazards related to this occupation and the ICPs adopted by the veterinarians in the study area in their day-to-day work. The present study revealed that most of the veterinarians worked for more than 50 hours a week, which is similar to that reported for Australian zoo veterinarians [24]. Despite awareness of issues, such as mental and physical stresses [25], awareness of biological health hazards, including mycotic infections, mange, swine erysipelas, anthrax and tuberculosis, brucellosis [13, 14, 26], leptospirosis [15, 27], Q-fever [16, 17], and *Toxoplasma gondii* (due to its teratogenic potential among working pregnant female veterinarians, if infected in the first trimester) [28], remains one of the prominent risks in this occupation. Furthermore, female veterinarians should be cautious before planning for pregnancy and throughout their pregnancy regarding the possibility of zoonosis infection while working in the field.

### 4.1. Individual Health

Animals are often uncooperative; hence, veterinarians are at higher risk of needlestick injury than other health care workers. Occupational needlestick injuries may cause transmission of infectious agents, which represents another major public health concern. The prevalence of needlestick injury (sharp management) among veterinarians in the same study area was previously found to be very high (80.9%) [5], and various studies have shown that it is the most common source of injury among these professionals. Transmission of infectious agents and other harmful agents through needlesticks during work hours is a serious public health concern [29]. In the present study, 28.5% and 3.4% of respondents mentioned needles containing antibiotics and vaccines (*brucella* and rabies vaccines), respectively, at the time of the puncture event. Similar findings were reported in a study conducted among veterinarians in Illinois [20], that is, needles contained antibiotics, vaccines, anesthetics, and animal blood at the time of the puncture event. A study conducted among American female veterinarians (*n* = 457) reported that 6% and 3% experienced accidental exposure to rabies and *Brucella abortus* vaccine, respectively [30], which is similar to the findings of the present study. A comparatively increased incidence of accidental exposure to vaccines due to needlestick injury was reported among Canadian veterinarians (26% (214/810)) [10]. This stark difference may be due to differences in animal practices. Animal vaccines are not tested for safety in humans, so their adverse effects remain unexplored, which may affect the health of veterinarians.

#### 4.1.1. Routine Medical Health Checkup

Despite routine handling of diseased and potentially infectious animals, infectious material, and work-related hazards, such as mental stress, 36.4% of the respondents did not undergo a routine medical health checkup in the past. In contrast, 35.8% of the respondents underwent a routine medical health checkup during the past 1 year, which is lower than the value in a study conducted among veterinarians from Western countries, where 87% consulted physicians within the previous 30 months [20]. This difference may be explained by the awareness of occupational hazards and the need for health checkups in Western countries as a job profile.

#### 4.1.2. Ailments at the Time of Interview

At the time of the interview, half of the veterinarians had no ailments, whereas the remaining half of the respondents suffered from different ailments, such as anxiety (9.5%), diabetes (4.7%), enteric disorders (6.4%), and hypotension or hypertension (14.9%). Veterinarians and associated staff may be at higher risk for allergen exposure from both the animals (fur, dandruff, urine, etc.) that they treat and some of the therapeutic agents that they use in their daily practice [4]. Among the half of the respondents who suffered from ailments, 10.4% suffered from allergies, that is, fur/dandruff (13.1%), latex gloves (1.6%), chemicals/drug (4.9%), vaginal secretion (1.6%), and amniotic fluid (1.1%). Allergies to body fluids, hair, dander, latex, and chemical allergens were also reported among Canadian veterinarians [10]. Dust and animal dung represent known allergic health hazards, whereas allergic rhinitis [31, 32] and conjunctivitis were the most commonly reported allergic conditions in animal-related occupations [33]. In the present study, no individual reported allergies due to antimicrobials, whereas Lessenger [20] reported that 12% of veterinarians suffered from the specific allergy. However, 12.5% of the respondents' allergies were so severe that they sought medical advice to control their allergies. The prevalence of allergies increased with the length of occupational exposure, and female veterinarians were more likely to develop allergies than male veterinarians [10, 11].

Despite the high risk for zoonotic infection, only 27% of the veterinarians were cautious about screening for zoonotic diseases. The majority (59.8%) of the respondents did not undergo zoonotic disease screening in the past two years. Our findings are similar to those in a study conducted in the Northern part of the country (Punjab state) for brucellosis exposure among animal handlers, para veterinarians, and veterinarians, which reported that amongst the different tehsils (subdistricts), the veterinarians from Khanna were least likely to have been tested for brucellosis [34]. Screening of zoonotic diseases is very important for the health of the veterinarians; in a survey conducted among Australian veterinarians, wherein 44.9% (*n* = 344) reported acquiring a zoonotic disease throughout their career [35], a higher percentage of positive for zoonoses reported among Australian veterinarians may be because of a higher percentage of screening of zoonotic diseases due to more awareness and availability of zoonotic diseases screening facility. A survey conducted among researchers in a veterinary facility in Nigeria reported a current knowledge of laboratory biosecurity and biosafety is limited [36]. This report is consistent with the finding that humans were exposed to biological hazards in this occupational setting largely due to known or unknown exposure to live animals or animal products. In particular, farm animal veterinarians (large animal veterinarians) are threefold more likely to contract zoonotic diseases than those working in other veterinary practices [20].

#### 4.1.3. Prophylactic Vaccination

If ignored, bite injuries from animals may cause fatal diseases [11], such as rabies. Rabies is endemic in India, and India is a leading country of rabies-associated deaths. The incidence of human rabies in India is 20,565 per year, and dog bites (96.2%) are the main cause [37]. However, half (49.3%) of the veterinarians in the present study population did not receive common vaccinations, such as rabies and tetanus. Potentially due to increased awareness among wildlife veterinarians, 62.9% of these veterinarians had received prophylactic vaccination for rabies in the country [38]. Three doses for pre-exposure prophylaxis for rabies at days 0, 7, and 21 or 28 are recommended for veterinarians and animal handlers in India [39]. A study conducted among US veterinarians in Oregon found that 13.9% had never been vaccinated for rabies [40], and a lower percentage of prophylactic vaccination might be due to awareness about zoonoses among veterinarians from developed countries than developing countries. Despite the high risk of fatal zoonotic diseases among these professionals, internationally conducted studies among veterinarians have demonstrated a low rate of rabies immunization. For example, half (47%) of Illinois veterinarians received a tetanus vaccine in the last 18 months, and other studies have reported a low rate of rabies immunization despite bite injuries [20].

### 4.2. Infection Control Practices (ICPs)

#### 4.2.1. Use of Personal Protective Equipment (PPE)

e USA [21], 18 of 71 (25.4%) human infections occurred among veterinary personnel. The contraction of zoonoses by veterinarians and associated staff, for example, highlights the need for ICPs in veterinary medicine such as using PPE while handling animals to significantly minimize the risk of contracting zoonosis. In the present study, half (49.0%) of the respondents wore aprons, facemasks, and gloves as a precaution to avoid zoonoses, which is less than the Australian veterinarians (75.0%) as they used “adequate” PPE to avoid zoonoses [35]. However, 32.4% of the respondents only wore gloves, and 8.4% of the respondents worked in the field without PPE. Despite the high risk of contracting zoonotic diseases from wild animals, a comparatively increased percentage (16.7%) of Indian wildlife veterinarians (*n* = 54) reported working in the field without taking any precautions or using PPE [38]. The incidences of fatal diseases, such as leptospirosis and bird flu, are reduced when proper PPE is used [15]. The higher percentage of respondents taking precautions in the present study may be due to increasing awareness among the veterinarians about biological hazards/zoonotic diseases. Similarly, Wright et al. [21] has also reported that PPE could prevent zoonoses to some extent among large-animal veterinarians in the USA [21]. We did not collect information on ICPs based on clinical cases. However, a study conducted among Australian veterinarians revealed that 60–70% did not use PPE while treating respiratory and neurological cases, whereas 40–50% of the respondents did not use PPE while treating gastrointestinal and dermatological cases [35]. A study focused exclusively on risk factors for *Brucella* infection among veterinarians showed a need for investigation to clarify the effectiveness of PPE to reduce *Brucella* infection [14]. A study also confirmed that brucellosis was found a common occupational zoonosis among veterinary personnel (*n* = 279).

#### 4.2.2. Personal Hygiene

Personal hygiene is an important measure for reducing the risk of zoonosis and other diseases along with early recognition of infected animals, proper animal handling, and basic biosecurity precautions. Hand-to-hand contact has been reported as the most common form of disease transmission, so consistent hand hygiene is very important in reducing the risk of disease spread [41]. Veterinarians are at risk of methicillin-resistant *Staphylococcus aureus* (MRSA) infection [42], and handwashing can prevent such infections. Unwashed hands may pose a risk of nosocomial transmission among different patient species [21]. Proper handwashing before and after animal contacts and handling blood, body fluids, secretions, excretions, and equipment or articles is a significant tool to reduce the number of transient organisms. In the present study, 40.2% and 27.9% of the respondents washed their hands between patients and once after finishing work, respectively. Veterinarians from the USA reported lower rates of washing their hands between patient contacts (18%) compared with the present study population [21]. In one UK study, handwashing practices by doctors were less common (8.1% and 51.4%) before and after patient contacts than those by nurses (20.4% and 60.1%) [43]. Wearing gloves reduces the transmission of organisms by providing barrier protection but is not a substitute for hand hygiene [44].

### 4.3. Interprofessional Collaboration

#### 4.3.1. Communication between Physicians and Veterinarians

Considering the ideology of “One Health,” data were obtained to understand communication between veterinarians and physicians. Surprisingly, in the present survey, the majority (70.2%) of respondents reported a lack of communication and mentioned that physicians never contacted them regarding zoonotic diseases. In contrast, 55.6% of the respondents mentioned that they had never contacted physicians regarding zoonoses. The findings of the present study were consistent with the findings of a study conducted among veterinarians (*n* = 370) in King County, as they have found that >50% of the surveyed veterinarians had never spoken to physicians about zoonoses [22]. A study conducted in the USA revealed that 100% of physicians and 97% of veterinarians claimed to never or rarely contacted individuals from other professions when seeking advice on zoonotic disease risks or cases [21].

Veterinarians must also be aware of their responsibility to protect colleagues and associated staff, particularly nontechnical staff who may not know or be unaware of how to recognize and adequately protect themselves from zoonoses. A study conducted in Punjab state India has underscored that lack of awareness about zoonotic diseases among animal handlers compared to paraveterinarians and veterinarians places them at higher risk to contract *Brucella* organism [34]. In Australia, veterinarians are legally required to provide this information [24]. Zoonotic diseases pose risks of substantial liability to veterinary practice owners from both legal and occupational health perspectives in the USA [21]. The present study reported that 54.2% and 38.3% of the total respondents never and rarely discussed zoonoses among their client population and subordinates to educate or train them, respectively. The majority of King County veterinarians (77%) agreed that it was very important to educate clients on zoonotic diseases prevention, but only 43% of the respondents-initiated discussions about zoonotic diseases prevention with clients on a daily basis, and 57% mentioned having client educational materials on zoonotic diseases in their practices [22].

Most of the respondents (87.7%) showed an urgent need for the inclusion of OHS practices in continued veterinary education (CVE) programs. Although the adoption and implementation of specific infection control guidelines might increase work hours, it will be helpful to increase client trust and economic performance [21].

### 4.4. Awareness and Knowledge about Occupational Health and Safety (OHS)

Approximately 34.9% and 47% of the veterinarians were aware of physical and biological hazards, whereas few (3.1% and 3.6%) were aware of chemical hazards and allergies, respectively, in their occupation. Similar results were observed among zoo veterinarians who were more likely to report inadequate knowledge of occupational hazards [4]. Surprisingly, knowledge of occupational hazards was inadequate among full-time American zoo veterinarians [45].

## 5. Limitations of the Study

The present study was conducted under a cross-sectional design and recorded the responses of practicing veterinarians through a set of questionnaires in a self-assessment manner. The information provided by the study subjects on past events can be a potential source of recall bias.

## 6. Conclusions

As per study observations, subjects were less attentive to their own health and lacked awareness and knowledge on OHS; this may pose a risk to Indian veterinarians for contracting the zoonotic disease(s). It is practically impossible to completely prevent exposure to biological hazards. However, education and training on zoonoses and implementation of effective ICPs can prevent biological hazards to a certain extent by identification of infected animals (for isolation), appropriate use of PPE, proper handling and housing of animals, and personnel hygiene (such as proper handwashing) among veterinarians. To create awareness regarding prophylactic vaccination, veterinary medical schools should vaccinate veterinary students at the time of enrollment based on US veterinary school regulations. Guidelines in the “Guidebook on Adult Immunization in Occupational Health Settings (2020)” should be followed by working field veterinarians, and employers should adhere to these guidelines. Different countries have formulated and published infection control-related guidelines for veterinarians in their countries according to the field condition, such as the Compendium of Veterinary Standard, Precautions for Zoonotic Disease Prevention in Veterinary Personnel, and National Association of State Public Health Veterinarians. Veterinary Infection Control Committee 2010 published in the *Journal of American Veterinary Medical Association* (JAVMA) as a guide for precautionary measures in America. The Australian Veterinary Association (AVA) has formulated and published a guide for the use of PPE; however, such comprehensive guidelines for Indian veterinarians are lacking. Moreover, routine health checkups, regular deworming of veterinarians, and medical record-keeping (including zoonoses screening) by the employer with monthly progress reports would help improve the quality of life of these professionals. The curricula in veterinary schools in the country should also emphasize occupational health and safety (OHS) practices. Standard protocols/guidelines for ICPs in veterinary clinics/dispensaries are lacking, and minimal emphasis is placed on measures to reduce the prevalence of zoonotic transmission, which is underscored by the lack of research published to date.

## Figures and Tables

**Table 1:** Needle-stick-related information among Indian veterinarians.

Description	*n* (%)	95% CI
*Needlestick injury (n* *=* *562)*		
Yes	455 (80.9)	(77.49, 83.99)
No	107 (19.0)	(16.00, 22.50)
*Material present at the time of puncture event (n* *=* *536) *		
Brucella vaccine	3 (0.6)	(0.1, 1.74)
Rabies vaccine	15 (2.8)	(1.6, 4.63)
Nonspecific drug	322 (60.1)	(55.8, 64.14)
Antibiotics	153 (28.5)	(24.8, 32.52)
Anthelmintic	2 (0.4)	(0.1, 1.46)
Steroids	8 (1.5)	(0.7, 2.99)
Not applicable	33 (6.2)	(4.4, 8.56)

**Table 2:** Status of medical health checkups among Indian veterinarians.

Description	*n* (%)	95% CI
*Time when last routine medical health checkup was performed (n* *=* *558)*		
One month	10 (1.8)	(0.9, 3.34)
1 year	190 (34.0)	(30.2, 38.09)
2 years	78 (14)	(11.3, 17.13)
3–5 years	77 (13.8)	(11.1, 16.94)
No medical checkup	203 (36.4)	(32.4, 40.46)

*Ailments at time of the interview (n* *=* *550) *		
Anxiety	52 (9.5)	(7.2, 12.22)
Allergies	57 (10.4)	(8, 13.22)
Diabetes	26 (4.7)	(3.2, 6.88)
Enteric disorder	35 (6.4)	(4.5, 8.76)
Hypotension and hypertension	82 (14.9)	(12.1, 18.15)
Hypercholesterolemia	9 (1.6)	(0.1, 3.15)
No ailments	270 (49.1)	(44.9, 53.27)
Other	19 (3.5)	(2.2, 5.39)

*Allergies at time of the interview (n* *=* *550) *		
Fur/dandruff	72 (13.1)	(10, 16.2)
Latex gloves	9 (1.6)	(0.8, 3.15)
Chemicals/drugs	27 (4.9)	(3.3, 7.09)
Vaginal secretion	9 (1.6)	(0.8, 3.15)
Amniotic fluid	6 (1.1)	(0.4, 2.44)
Any other	8 (1.5)	(0.6, 2.92)
No allergy	419 (76.2)	(72.4, 79.55)

*Sought medical attention for your allergy ailments during the last 2 years (n* *=* *553)*		
Yes	69 (12.5)	(9.9, 15.52)
No	257 (46.5)	(42.3, 50.65)
Not required	175 (31.6)	(27.9, 35.65)
Do not remember	52 (9.4)	(7.2, 12.16)
4 months	66 (11.8)	(9.3, 14.72)
6 months	70 (12.5)	(9.9, 15.5)
1 year	106 (18.9)	(15.8, 22.36)
No routine deworming	319 (56.9)	(52.7, 60.9)

*Screening for any zoonotic infection during the last 2 years. (n* *=* *562) *		
Yes	152 (27.0)	(23.4, 30.77)
No	337 (59.8)	(55.6, 63.72)
Facility not available	67 (11.9)	(9.4, 14.84)
Not interested	6 (1.3)	(0.6, 2.85)

*If yes, then specify the zoonotic diseases (n* *=* *552) *		
Brucellosis	148 (26.8)	(23, 30.67)
Toxoplasmosis^#^	1 (0.2)	—
Leishmaniasis^#^	1 (0.2)	—
Ringworm (dermatophytosis)^#^	1 (0.2)	—
Tuberculosis^#^	1 (0.2)	—
Not applicable	400 (72.5)	(68.5, 76.03)

*Status of your prophylactic vaccination during the past 2 years (n* *=* *560)*		
Rabies	221 (39.5)	(35.5, 43.58)
Tetanus	62 (11.1)	(8.7, 13.97)
Hepatitis A/B^#^	1 (0.2)	—
No vaccine received	276 (49.3)	(45.1, 53.42)

^#^CI not calculated due to insufficient *n*.

**Table 3:** Infection control practices (ICPs) among Indian veterinarians.

Description	*n* (%)	95% CI
*Type of precaution followed to avoid zoonotic hazards (n* *=* *561)*		
No precaution	47 (8.4)	(6.3, 11)
Apron only	57 (10.2)	(7.9, 12.97)
Gloves only	182 (32.4)	(28.7, 36.44)
Apron, facemask, and gloves	275 (49.0)	(44.9, 53.15)
*Method of handwashing with soap and disinfectant (n* *=* *562)*		
Between patient examinations	226 (40.2)	(36.2, 44.33)
Once after completing work	157 (27.9)	(24.3, 31.8)
Seldom	10 (1.8)	(0.9, 3.31)
Never^#^	1 (0.2)	—
Before eating only	35 (6.2)	(4.5, 8.58)
Frequently	69 (12.3)	(9.8, 15.28)
Before and after work	64 (11.4)	(9, 14.31)

^#^CI not calculated due to insufficient *n*.

**Table 4:** Interprofessional collaboration to prevent zoonoses among Indian veterinarians and physicians.

Description	*n* (%)	95% CI
*Physicians (human) consulted you for advice on zoonotic diseases (n* *=* *560)*		
Several times/week	15 (2.7)	(1.6, 4.43)
Several times/month	22 (3.9)	(2.5, 5.93)
Rarely	130 (23.2)	(19.9, 26.9)
Never	393 (70.2)	(66.2, 73.82)
Several times/week	8 (1.4)	(0.6, 2.89)
Several times/month	10 (1.8)	(0.9, 3.35)
Several times/year	26 (4.7)	(3.1, 6.81)
Rarely	203 (36.5)	(32.6, 40.6)
Never	309 (55.6)	(51.4, 59.66)

**Table 5:** Knowledge about occupational health and safety (OHS) among Indian veterinarians.

Description	*n* (%)	95% CI
*Awareness about different occupational hazards in veterinary medicine (n* *=* *478)*		
Physical hazards	167 (34.9)	(30.8, 39.33)
Biological hazards	95 (19.9)	(16.5, 23.71)
Chemical hazards	15 (3.1)	(1.8, 5.18)
Allergies	17 (3.6)	(2.2, 5.68)
Psychological/mental	3 (0.6)	(0.1, 1.95)
All of the above	165 (34.5)	(30.3, 38.9)
None of the above	16 (3.3)	(2, 5.43)
*Source of information for occupational hazards (n* *=* *551)*		
News bulletin	78 (14.2)	(11.4, 17.34)
Internet	220 (39.9)	(35.9, 44.08)
Conference	39 (7.1)	(5.2, 9.57)
Meeting/training	214 (38.8)	(34.8, 42.98)
*Urgent need to include occupational hazards in CVE program (n* *=* *554)*		
Yes	486 (87.7)	(84.7, 90.21)
No	20 (3.6)	(2, 5.56)
Cannot say	27 (4.9)	(3.3, 7.04)
Do not know	21 (3.8)	(2.4, 5.78)
*Discussions among your client population/subordinates about zoonotic diseases (n* *=* *557)*		
Several times/day	70 (12.6)	(10, 15.61)
Daily	76 (13.6)	(11, 16.77)
Weekly	36 (6.5)	(4.6, 8.86)
Occasionally	315 (56.6)	(52.4, 60.61)
Never	60 (10.8)	(8.4, 13.65)

## Data Availability

The [quantitative data] data used to support the findings of this study are included within the article.
